# Regulation of the Proteolytic Activity of Cysteine Cathepsins by Oxidants

**DOI:** 10.3390/ijms21061944

**Published:** 2020-03-12

**Authors:** Gilles Lalmanach, Ahlame Saidi, Paul Bigot, Thibault Chazeirat, Fabien Lecaille, Mylène Wartenberg

**Affiliations:** 1 Université de Tours, 37000 Tours, France; ahlame.saidi@univ-tours.fr (A.S.); paul.bigot-2@etu.univ-tours.fr (P.B.); thibault.chazeirat@etu.univ-tours.fr (T.C.); fabien.lecaille@univ-tours.fr (F.L.); mylene.wartenberg@univ-tours.fr (M.W.); 2 INSERM, UMR1100, Centre d’Etude des Pathologies Respiratoires, 37000 Tours, France

**Keywords:** cathepsin, chronic obstructive pulmonary disease (COPD), cysteine, cysteine protease, lung inflammation, oxidation, proteolysis, thiol

## Abstract

Besides their primary involvement in the recycling and degradation of proteins in endo-lysosomal compartments and also in specialized biological functions, cysteine cathepsins are pivotal proteolytic contributors of various deleterious diseases. While the molecular mechanisms of regulation via their natural inhibitors have been exhaustively studied, less is currently known about how their enzymatic activity is modulated during the redox imbalance associated with oxidative stress and their exposure resistance to oxidants. More specifically, there is only patchy information on the regulation of lung cysteine cathepsins, while the respiratory system is directly exposed to countless exogenous oxidants contained in dust, tobacco, combustion fumes, and industrial or domestic particles. Papain-like enzymes (clan CA, family C1, subfamily C1A) encompass a conserved catalytic thiolate-imidazolium pair (Cys25-His159) in their active site. Although the sulfhydryl group (with a low acidic pKa) is a potent nucleophile highly susceptible to chemical modifications, some cysteine cathepsins reveal an unanticipated resistance to oxidative stress. Besides an introductory chapter and peculiar attention to lung cysteine cathepsins, the purpose of this review is to afford a concise update of the current knowledge on molecular mechanisms associated with the regulation of cysteine cathepsins by redox balance and by oxidants (e.g., Michael acceptors, reactive oxygen, and nitrogen species).

## 1. Cysteine Cathepsins

**Figure 1:**
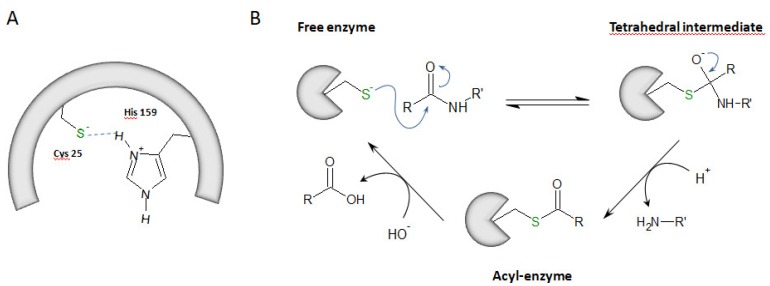
The hydrolysis mechanism via cysteine cathepsins. (**A**) Catalytic dyad: Cys25 (papain numbering)/His159. Its ionic form is shown, i.e., thiolate (Cys-S^−^)/imidazolium (His-ImH^+^). (**B**) The catalytic mechanism: a cartoon representation.

Proteases are classified into six distinct types according to residues essential for their enzymatic activity and their catalytic mechanism: serine proteases, acid (aspartate and glutamate) proteases, cysteine proteases, metalloproteases, and threonine proteases [1,2]. Cysteine proteases are widely expressed in animals, plants, fungi, parasites, bacteria or viruses [3,4]. Among them, cysteine cathepsins B, C, F, H, K, L, O, S, V, W and X (clan CA, family C1, subfamily C1A) are structurally related to papain (from *Carica papaya*) [5,6]. Primarily, cysteine cathepsins are ubiquitous lysosomal proteases that are active at acidic pH and are rapidly inactivated at neutral pH (except cathepsin S, CatS) [7]. In addition, some cathepsins (e.g., cathepsins B, L, K, H and S) could be secreted into the extracellular medium by alveolar macrophages, epithelial cells, pneumocytes or fibroblasts [8,9]. Alternatively, under specific pathophysiological conditions, truncated cathepsins could be targeted to mitochondria or found in the cytosol before being imported into the nuclear compartment, where they can even function as active proteases [10,11]. This unexpected cellular roadmap was nicely summarized elsewhere [12]. The half-life of cysteine cathepsins may vary from a few minutes to several hours depending on the cellular environment [13,14]. Besides housekeeping recycling and degradation of proteins within acidic compartments, they participate in specific biological processes, such as the maturation of some prohormones, apoptosis, antigen presentation or remodeling of the extracellular matrix and basement membrane [15]. They are also involved in pathological processes (e.g., rheumatoid arthritis, osteoporosis, asthma, cancer, and inflammation). Accordingly, some cysteine cathepsins are considered as valuable therapeutic targets, and pharmacological inhibitors of these proteases are currently in clinical trials [16,17,18,19]. Cysteine cathepsins are synthesized as pre-proenzymes, and their corresponding proregions participate in folding and enzyme stability (for review: Reference [6]). The processing of the mature active form occurs by cleavage and release of the propeptide. Propeptides may also act as competitive inhibitors of their parent mature and active enzyme (catalytic domain). In addition, cysteine cathepsins can be transcriptionally modulated as well regulated by pH, temperature, oxidation, glycosaminoglycans (GAGs) and by endogenous inhibitors [17], including the cystatin family (i.e., stefins, cystatins, and kininogens) [3,20,21]. Cysteine cathepsins are monomeric enzymes (22–28 kDa range), with the exception of cathepsin C, which is a tetrameric molecule (circa 200 kDa) [22]. The archetypal structural shape of cathepsins corresponds to a left (L) domain and a right (R) domain that are of similar size. Both CatB and CatX share an additional structure called the occlusion loop that drives their exopeptidase activity [23,24]. Their catalytic mechanism requires the presence of a catalytic dyad composed of Cys25 (papain numbering; subsequently used throughout the text to designate the catalytic cysteine within the active site) and His159. At pH 3.5–8.0, this dyad is under its ionic form, i.e., thiolate/imidazolium (Cys-S^−^/His-ImH^+^), which results from the transfer of a proton from Cys to His. A third residue (Asn175) contributes to the catalytic mechanism by maintaining His159 via a hydrogen bond in the correct positioning [25]. The first step of the mechanism is a nucleophilic attack of the carbonyl group of the peptide bond by the thiolate group. It results in an anionic tetrahedral intermediate that forms an oxyanion, stabilized by a hydrogen bond between Cys25 and Gln19. Then, His159 loses its proton, leading to the release of the amine portion of the substrate and the subsequent contribution of a water molecule to the formation of a second intermediate acyl-enzyme. The ending deacylation step allows both the release of the carboxyl portion of the hydrolyzed substrate and recovery of the free enzyme [25,26] (Figure 1).

While the molecular mechanisms of regulation by their natural inhibitors have been exhaustively studied, little is known about how the enzymatic activity of cathepsins is modulated during exposure to oxidants, as well during the pathophysiological oxidant/antioxidant imbalance associated with oxidative stress. Indeed, besides pH values ranging from basic to highly acidic, redox conditions from oxidizing conditions (in the compartments of the secretory pathway and extracellular space) to reducing conditions (within endo-lysosomal compartments) are crucial determinants of proteolytic activity [27]. More particularly, there is only scarce information on the regulatory mechanisms of pulmonary cathepsins, while the lung is a first-line organ directly exposed to many exogenous oxidants contained in dust, combustion fumes, and industrial or domestic particles. Specifically, COPD (chronic obstructive pulmonary disease) is a lung pathology that is characterized by progressive and irreversible airflow blockade. There is both an obstruction of the airways (chronic bronchitis) and destruction of the alveolar walls (emphysema), aggravated by exacerbation phases (bacterial or viral infections). The etiology of COPD (the 3rd leading cause of death in the world in 2030) is primarily based on the inhalation of particles, irritants, and numerous oxidant chemicals found in cigarette smoke (circa 10^20^ oxidant molecules per puff). In this review, we will present a chapter dedicated to lung cysteine cathepsins. Next, we will focus on basic mechanisms of oxidation of thiol functions followed by an update of the current knowledge of the regulation of cysteine cathepsins by oxidants. 

## 2. Cysteine Cathepsins in Pulmonary Diseases

Cysteine cathepsins are expressed in most lung tissues, and some of them (cathepsins B, L, H and X) are ubiquitously distributed in epithelial cells, macrophages as well fibroblasts (for review: Reference [8]). In contrast, CatW is predominantly expressed in cytotoxic T-lymphocytes and natural killer cells, hence it is also known as lymphopain [28]. Cysteine cathepsins probably play an essential role in lung homeostasis. Besides metalloproteinases such as MMP-2 (gelatinase A), MMP-9 (gelatinase B) and stromelysin [29,30,31], they participate in the remodeling and degradation of ECM (extracellular matrix) and BM (basement membrane) components [6]. CatK displays the unique property to cleave type I and II collagens in their triple helices (for review: Reference [32]), while cathepsins B, F, K, L, S and V have a potent elastinolytic activity and may degrade elastin fibers [6,33]. Both CatK and CatS are primarily implicated in extracellular degradation, whereas CatV is mainly involved in lysosomal digestion [34]. CatH is involved in the maturation of SP-B, a hydrophobic surfactant protein involved in the reduction of surface tension, and thus has a beneficial role in pulmonary homeostasis [35]. Moreover, CatH participates in the maturation of surfactant protein SP-C [36,37]. CatS was found in the cilia of conducting airway cells, suggesting a possible role in cilial motility [38]. 

The putative importance of cysteine cathepsins in lung diseases has emerged for two decades (for review: References [9,39,40]). Silicosis is a professional pneumoconiosis caused by chronic inhalation of crystalline silica, leading to progressive fibrosis and tissue damage [41,42]. Active cathepsins B, H, K, L and S were found in the bronchoalveolar lavage fluids of patients with silicosis associated with a protease/antiprotease imbalance in favor of cathepsins [43]. Based on an animal model of silicosis, it was proposed that CatK could have a beneficial role in preventing the increase of matrix deposition and play a protective role against fibrotic processes [44]. Also, C57BL/6 mice that are susceptible to silicosis display a lower CatK expression than resistant BALB/c mice [45]. Idiopathic pulmonary fibrosis (IPF) is the most relentless form of idiopathic interstitial pneumonia, with a median survival of 3 years after diagnosis [46,47]. Transforming growth factor-1 (TGF-β1) plays a central role in fibrogenesis by stimulating the activation, proliferation, and differentiation of fibroblasts into α-smooth muscle actin (α-SMA)-expressing myofibroblasts that secrete excessive amounts of ECM components [48]. We showed that CatB participates in the TGF-β1-driven differentiation of lung fibroblasts via the intracellular processing of pro-TGF-β1 to its 25-kDa mature form. Afterwards, secreted TGF-β1 could trigger the Smad 2/3 signaling pathway, thus initiating myofibrogenesis [49]. In the murine model of bleomycin-induced pulmonary fibrosis, CatK deficiency associates with severe lesions; CatK inactives proteolytically TGF-β1, thereby supporting that CatK contributes to airway structural integrity [50,51]. Nevertheless, the situation remains highly complex, and diverse data suggest adaptable roles of cathepsins K, S, B and L in TGF-β signaling depending on fibrotic tissues [52]. Cystic fibrosis (CF) is a rare genetic disorder, which leads to an increase in mucus viscosity and its accumulation in the airways, providing a fertile ground for opportunistic pathogens associated with chronic infections and inflammation [53]. In CF patients, the alveolar epithelial fluids are acidified (circa pH 6), which ensures favorable conditions for cathepsin activity [54]. A recent study reported that urinary CatB level in children with cystic fibrosis is significantly increased [55]. CatS is upregulated in the lungs of CF patients and was reported as a potential marker of inflammation during cystic fibrosis [56]. In addition, instillation of CatS into the lungs of wild-type mice results in inflammation, lung remodelling, and upregulation of mucin expression, while CatS inhibition leads to a reduction in airway inflammation and mucin expression. Taken together, it has been proposed that CatS plays a critical role in the pathogenesis of cystic fibrosis and some CF-like lung diseases, highlighting CatS as a valuable therapeutic target [57]. Nevertheless, cathepsins B, H, K, L, and S levels remain unchanged in the sputum of CF patients infected or not by *Pseudomonas aeruginosa*, suggesting that they are not markers of bacterial infection [58]. Moreover, cathepsins B, L and S may contribute to the dysfunction of the inflammatory response by proteolytic inactivation of AMPs (antimicrobial peptides and proteins), such as β-defensins-2 and -3, lactoferrin, SP-A and LL-37 [59,60,61,62,63]. CatL is also capable of degrading α1-antitrypsin, a member of the serpin family, thereby promoting the uncontrolled proteolytic activity of serine proteases [64]. Asthma is a chronic inflammatory lung disease whose main features are hyperreactivity of the airways and hypersecretion of mucus leading to airflow obstruction. Asthma is associated with a recruitment of immune cells (e.g., mast cells or eosinophils) that secrete pro-inflammatory cytokines [65,66]. Chronic inflammation will lead to proliferation of smooth muscle cells, subepithelial fibrosis, and neovascularization. A significant increase of CatS was observed following inhalation of aerosolized ovalbumin in an induced-asthma animal model [67]. Conversely, pharmacological inhibition of CatS reduces the allergic reaction induced by ovalbumin, suggesting that CatS is involved in the pathogenesis of asthma and that CatS could be used as a biomarker for disease progression and response to treatment [68]. Also, CatS is involved in both the release of antigenic peptides and in the maturation of MHC class II molecules and is essential for the generation of the invariant chain (Ii) [69]. Together with CatF from alveolar macrophages [70], the inhibition of CatS could be beneficial in the treatment of pathologies with an inappropriate or excessive immune response such as asthma [71]. As reported in the previous paragraph, COPD that corresponds to both chronic bronchitis and emphysema is essentially due to exposure to cigarette smoke, and there is some evidence that proteases and oxidants act synergistically to mediate injury and promote inflammation and uncontrolled remodelling of the small airways. Among diverse reactive oxygen species, high levels of hydrogen peroxide (H_2_O_2_), a potent marker of oxidative stress, were detected in the expired condensates of COPD patients [72]. Overexpression of CatS was observed in mouse models of experimental emphysema induced by IL-13 or IFN-γ; conversely, CatS inhibition by pharmacological inhibitors reduces significantly the severity of emphysema and inflammation [7,73,74]. Also, cigarette smoke enhances the production of IL-18 (an IFN-γ inducing factor), and induces CatS expression in lung macrophages of cigarette smoke-exposed mice [75]. Recently, we demonstrated that CatS expression, as well its enzymatic activity, is more elevated in current smokers (both non-COPD and COPD patients) compared to never-smokers, and these correlate positively with smoking history [76]. Exposure of primary human bronchial epithelial cells to cigarette smoke extract (CSE) triggers the activation of purinergic P2X7 receptors, which in turn drive CatS upregulation [76]. Despite a marked increase of oxidants associated with cigarette smoke and a subsequent oxidant/antioxidant imbalance, overall data suggest that CatS may contribute, in conjunction with other proteases, to elastinolysis and subsequent parenchymal destruction in emphysema [77].

Besides pulmonary diseases, cysteine cathepsins are involved in various pathologies for which the role of oxidative stress is firmly established. For example, a decrease of uteroplacental blood flow and oxygen availability associated with an increase of oxidative stress was reported during preeclampsia. Cysteine cathepsins were identified as putative worthy players of the dysfunctional placental vascular remodeling that occurs during preeclampsia [78]. Also, these proteases participate to inflammatory activation, cholesterol metabolism, neovascularization, and cell signaling, as well tissue fibrosis, and contribute to cardiovascular diseases (CVD); among other regulatory factors, cysteine cathepsins are regulated by oxidative stress during the development of CVD (see for review: Reference [79]). Otherwise, cathepsin-cleaved Bid promotes apoptosis in human neutrophils via oxidative stress-induced lysosomal membrane permeabilization [80]. Impaired autophagy and lysosomal degradation are linked with increased oxidative stress, triggering degenerative changes in the aging neurons [81]. On the other hand, deletion of CatB significantly reduced the generation of ROS and neuroinflammation and improved cognitive weakening during aging [82], whereas oxidative stress-mediated activation of NLRP3 inflammasome in microglia occurs through upregulation of CatB activity [83]. Accordingly, blood-brain barrier-permeable inhibitors for CatB may represent a new therapeutic strategy against brain aging and neurological diseases including Alzheimer’s disease, Parkinson’s disease, stroke, and persistent pain [84].

## 3. Inactivation of Cysteine Cathepsins by Oxidants

### 3.1. Oxidation of Thiols: A General Context

**Figure 2:**
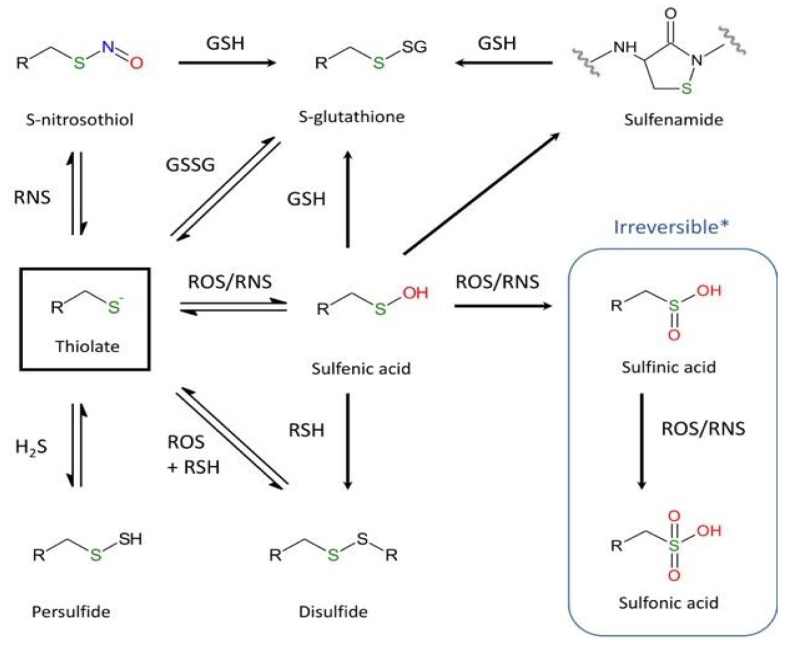
Major oxidative and nitrosative post-translational modifications of cysteine. (R = –NH–C_α_H–CO–) (* except the specific reduction of 2-Cys peroxiredoxins by sulfiredoxin [94,95]).

Although cysteine accounts for only 2% of total protein residues in mammals, it plays a crucial role in the function and structure of proteins. As mentioned by Poole [85], of the twenty common proteinogenic amino acids, perhaps the most intriguing and functionally diverse is cysteine. The sulfhydryl group, which is sensitive to chemical modifications, may exist at different oxidative states [86,87,88]. Differences in thiol reactivity mostly depend on the broad values of pKa (acid dissociation constant) (Figure 2). Even though pKa values of most thiol groups are *circa* pH 8.5 [85], pKa values of protein thiols can range from 2.5 to 12 [89,90,91,92]. Moreover, the thiol reactivity also relates to interactions with neighboring residues of the microenvironment. Redox modifications of Cys depends on the nature and the concentration of the reactive oxygen and nitrogen species (ROS/RNS) and can be divided into two general distinct oxidation products: reversible forms (e.g., intra- or inter-molecular disulfide bridges, sulfenic acid (R-SOH), or nitrosylated products) and irreversible forms, including sulfinic (R-SO_2_H) and sulfonic (R-SO_3_H) acids. The formation of sulfenic acid following exposure to oxidants such as H_2_O_2_ or HOCl is one of the most usual reversible reactions occurring in response to oxidative stress. Next, R-SOH can also react with reduced glutathione (GSH), leading to S-glutathionylation (R-SSG). On the other hand, the formation of the disulfide bridge, which is essential for the function and stability of numerous proteins, primarily depends on two mechanisms: a conventional oxidation-reduction reaction or the generation in the presence of ROS/RNS of a sulfenic acid that can react in a second step with another nearby cysteine to form a disulfide bridge [93]. 

**Figure 3:**
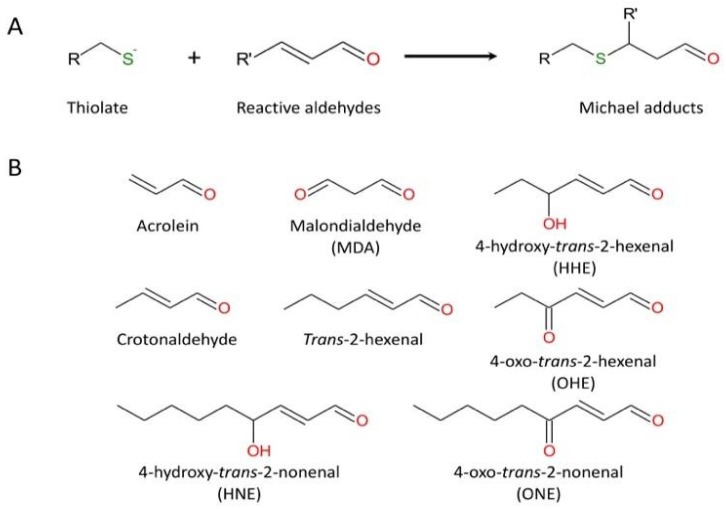
The reaction of cysteine (thiolate form) with representative unsaturated aldehydes. (**A**) general mechanism; (**B**) usual α, β-unsaturated aldehydes.

S-thiolation (i.e., the formation of reversible sulfenic acid and disulfide bridge) has been proposed as a temporary protective mechanism used by enzymes during oxidative stress to prevent irreversible changes in their active site [96,97]. However, sulfenic acid can be converted to sulfinic acid by nucleophilic attack of a peroxide species (H_2_O_2_ or ONOO^−^). For a long time, R-SO_2_H was considered as an artifact of protein purification, but there is increasing evidence that this hyperoxidation is not an odd event. A quantitative analysis of the soluble proteins of the rat liver reported that 5% of cysteinyl residues consist of sulfinic acid [98]. Due to its low acidic pKa (pKa ~2), R-SO_2_H exists exclusively in its sulfinate deprotonated form (R-SO_2_^−^), a weak nucleophile unveiling little reactivity in cells. Accordingly, sulfinic acids are considered stable compounds that cannot be reduced in the cellular environment, with the noticeable exception of the specific reduction of 2-Cys peroxiredoxins by sulfiredoxin [94,95]. Nevertheless, R-SO_2_H could be further oxidized and converted to sulfonic acid (R-SO_3_H) by strong oxidizing agents such as halogens, hydrogen peroxide, and nitric acid [89]. Also, the sulfhydryl group can react with α, β-unsaturated aldehydes (including acrolein, a major chemical of cigarette smoke) by Michael addition (Figure 3). In turn, this highly reactive adduct may react with a nearby amino group and generate an imine functional group (Schiff base) [99]. 

### 3.2. Cysteine Cathepsins and Oxidants

While many redox-enzymes use distinctively different cysteine redox-couples for exchange, electron, atom, and radical transfer reactions, cysteine cathepsins, which require a favorable reducing environment (redox potential circa −220 mV; [100]) for their activity, rely on reduced cysteine to catalyze hydrolytic reactions [101]. Thus the modification of the redox environment has been proposed as a control mechanism for regulating cysteine cathepsins activity [102]. Accordingly, the thiol group of the cysteine residue of the catalytic site of papain-related proteases (family C1) is particularly sensitive to oxidation and chemical modifications [86,103], in correlation with the low pKa value (pKa ~4/4.5) of the conserved nucleophile Cys25 [86]. However, direct evidence of the oxidative inactivation of endo-lysosomal cathepsins, as well of their secreted forms, remains currently incompletely investigated [104,105]. Nevertheless, the reactivity of Cys25 of cathepsins in the presence of oxidants was scrutinized in some previous articles under in vitro and in cellulo conditions, and diverse oxidation states of Cys25 were partially depicted.

#### 3.2.1. Inactivation by Reactive Nitrogen Species

Papain is inactivated via the nitrosylation of Cys25 or the formation of mixed disulfide bridges following exposure with NO donors [106,107,108,109]. Inactivation is time- and dose-dependent and reversible following the addition of reducing agents. The S-nitroso compounds (i.e., S-nitroso-N-acetylpenicillamine (SNAP) or S-nitrosoglutathione (GSNO)) appear to preferentially form mixed disulfides, whereas the non-thiol NO donors (such as nitrosonium tetrafluoroborate (NOBF4), (±)-(E)-2- (E)-hydroxyimino-6-methoxy-4-methyl-5-nitro-3-hexenamide (NOR-3) or nitroprusside (SNP) lead to a S-nitrosylation [103,107,108,109]. It was proposed that NO donors may lead to the formation of sulfenic acid due to a direct reaction with NO or by the hydrolysis of an S-nitrosothiol intermediate [110]. Nevertheless, the lack of clear evidence of the formation of sulfenic acid or S-nitrosothiol in response to NO donors primarily depends on their lability and technical difficulties to detect them by mass spectrometry. Moreover, it has long been accepted that the inactivation of thiol-containing enzymes by NO donors results from the formation of a reversible S-nitrosothiol, despite the presence of reversible mixed disulfides or sulfenic acid was reported in thiol-containing enzymes following exposure to NO donors [108,109,110,111]. Given this situation, the use of specific redox probes directed against different oxidized forms of cysteine have a utility to bypass these analytical inconveniences (see Table 1; for in-depth methodological details, see an outstanding overview of the chemical methods used to label and map cysteine oxidation [112]).

On the other hand, SIN-1 (3-morpholinosydnonimine), a peroxynitrite (ONOO^−^) donor and a harmful oxidant, led to the generation of sulfinic and/or sulfonic acids resulting in the irreversible inactivation of papain. Rauhala and coworkers proposed that the formation of reversible S-nitrosylation and/or mixed disulfides would have a protective role for papain by preventing its irreversible inactivation by other RNS [111]. Inactivation by NO donors was also reported for human cathepsins B [127,128,129,130] and K [110]. CatB was inactivated by nitrosylation in the presence of the NO donor; moreover, GSH encompassed a protective effect since the oxidation was higher in its absence [130,131,132]. Using as models two macrophage cell lines, it was shown that CatB was irreversibly inactivated by nitroxyl (HNO), probably via the successive formations of a N-hydroxysulfenamide intermediate and an irreducible sulfonamide [133,134]. Also, the presence of endogenous NO donors that can form reversible adducts with Cys was not sufficient to protect CatB from its inactivation by HNO [134]. Percival and collaborators demonstrated that CatK, which plays a critical role in bone remodeling, is also sensitive to oxidation by NO donors [110]. Although CatK has two free cysteinyl residues, only the active site Cys25 was impacted by NO donors [110]. The inactivation mechanism by NO donors, which relates to the redox status, depends on two distinct processes. In the presence of GSH, NO caused the accumulation of GSNO, then induced the establishment of a mixed disulfide bridge via the initial generation of a glutathione adduct. Conversely, in the absence of GSH, NO donors led to the irreversible oxidation of Cys25 (as sulfinic and sulfonic adducts) via an intermediate sulfenic acid. Interestingly, NO donors inhibit the autoprocessing of procathepsin K into its mature form [110]. 

#### 3.2.2. Inactivation by Reactive Oxygen Species

**Figure 4:**
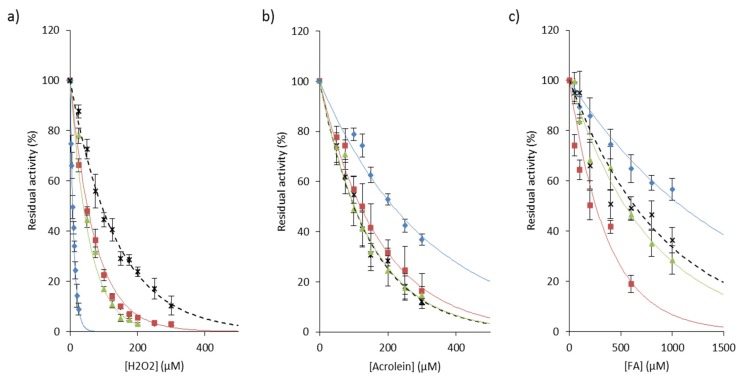
Inactivation of cathepsins B, K, L, and S by representative chemicals of cigarette smoke. Human cathepsins (1 nM) were incubated 20 min with increasing amounts of (**a**) H_2_O_2_ (0-300 µM), (**b**) acrolein (0–300 µM) or (**c**) formaldehyde (FA; 0–1000 µM), respectively, in 0.1 M sodium acetate buffer pH 5.5, containing 1 mM EDTA, 0.01% Brij 35 and 15 µM DTT. Residual activity was measured using Z-Phe-Arg-AMC (20 µM) as substrate (λ_ex_ = 350 nm, λ_em_ = 460 nm). Three independent assays were done in triplicate. Results were expressed as mean ± SEM. ♦: CatL; ■: CatK; ▲: CatB; **×**: CatS.

Exposure of papain to hydrogen peroxide predominantly led to the formation of a sulfenic acid that in turn reacted with adjacent free thiol and formed mixed disulfides [114,135,136,137,138]. The decrease in CatB activity has also been observed in a pheochromocytoma cell model (PC-12 cells) and in human embryonic kidney cells (HEK 293 cells) exposed to hydrogen peroxide [139,140]. On the other hand, following the addition of H_2_O_2_, Cys25 of CatK was found either in the form of sulfonic acid (~70% of –SO_3_H), either in the form of sulfenic acid (~30% of –SOH), which suggested that CatK activity could be partially recovered [141]. Also, analysis by redox sensing probes of the inactivation of CatS by H_2_O_2_ supported the formation of Cys25 sulfenic acid followed by a slower conversion to sulfinic acid [77]. This protective mechanism may explain the maintenance of cysteine cathepsin activity during emphysema [76] and inflammatory phenomena such as silicosis [43]. By broadening the proposal of Rauhala and coworkers [111], one could hypothesize that reversible inhibition of cathepsins by hydrogen peroxide may represent a complementary mechanism of protection against more harmful oxidants (i.e., hydroxyl radical, hypochlorous acid, or peroxynitrate). In addition, it was observed that monocytic cells secrete catalase that may protect extracellular enzymes against oxidative stress [142]. This proposal was confirmed by incubation with two distinct catalase inhibitors, KCN, a haem ligand and 3-amino-1,2,4-triazole [142]. Both GSNO and H_2_O_2_ inhibit the autocatalytic maturation of procathepsin K and inactivate the activity of the mature form in a time- and dose-dependent manner [102,133]. H_2_O_2_, but not ONOO^−^, hinders the autocatalytic maturation of procathepsin S [77]. Additionally, ROS impair the automaturation of procathepsin L and may disrupt its pro-autophagic function, while the processing of CatB zymogen is not blocked by ROS [143]. Fujii and collaborators showed that cathepsins B, L and S, but not acid cathepsins D and E, are irreversibly inactivated by singlet oxygen ^1^O_2_, suggesting that ^1^O_2_ reacts specifically with nucleophilic Cys25 of the active site [144,145]. Moreover, O_2_ and ^1^O_2_ can react with certain amino acids, peptides or proteins, leading to the formation of hydroperoxides that may inactivate in a time- and concentration-dependent manner cysteine cathepsins, but not non thiol-dependent cathepsins D and G [146,147,148]. The reaction between hydroperoxides and cysteine was evidenced by the loss of thiol function and the formation of a sulfenic intermediate. An analogous reversible inactivation by H_2_O_2_ was reported for cytosolic thiol-dependant caspases (peptidase family C14) [149]. It could be noticed that CatL is more sensitive to oxidation than CatB by hydroperoxides [146,147]. Also, CatS is less susceptible in the presence of H_2_O_2_ at acidic pH, while CatB is inactivated in a similar manner to CatK (Figure 4a). These results support that there is a difference in chemical reactivity of the invariant thiol group of their active site, although cathepsins L, S and K are enzymatically and structurally close. On the other hand, NADPH oxidase 2 (NOX2), which is found in the acidic compartments of antigen-presenting cells (i.e., macrophages and dendritic cells) and is involved in the synthesis of O_2_^−^, was reported to play a pivotal role during MHC class II antigen presentation through the redox control of antigen processing by cysteine cathepsins [150,151,152,153,154]. A new strategy that consists of generating ROS from redox cyclic compounds (RCCs) was proposed to target cysteine cathepsins [155]. 

#### 3.2.3. Redox Regulation

**Figure 5:**
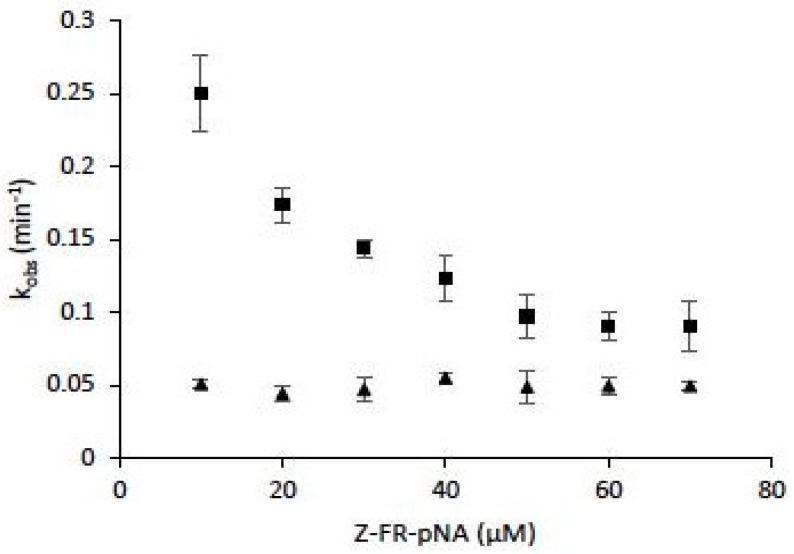
The effect of chondroitin-4-sulfate on human cathepsin K inactivation by oxidized glutathione (GSSG). Assays were performed both in the absence of chondroitin-4-sulfate (C-4S) (■) or in the presence of 0.15% (w/v) C-4S (▲) (*n* = 3). Human CatK (2 nM) was preincubated for 2 min at 30 °C in 0.1 mM sodium acetate buffer pH 5.0, 15 µM DTT, in the presence of 1 mM EDTA, 0.01 % Brij 35. The enzymatic activity was followed in 0.1 mM sodium acetate buffer pH 5.0, 20 mM GSSG, 15 µM DTT, 1 mM EDTA, 0.01 % Brij 35, in the presence of the chromogenic substrate Z-Phe-Arg-pNA (10–70 µM), at 30 °C. The progress curves were fitted to a single-exponential equation to obtain the observed first-order rate constant for inactivation (k_obs_) (Origin 6 software, Microcal Software, Northampton, MA, USA). Results were expressed as mean ± SEM.

Among effective and protective antioxidant systems [156], one of the most important is the GSH (reduced form)/GSSG (oxidized form) balance (GSH/GSSG ratio ~100 in cells) which has been implicated in immune and inflammatory responses, in mitochondrial breathing, in apoptosis, and in matrix remodeling (for review: Reference [157]). A GSH depletion was observed during pulmonary inflammations, and cigarette exposure causes an increase of epithelium permeability, associated with important changes of the GSH/GSSG system homeostasis of smokers (GSH/GSSG ratio ~10 in bronchoalveolar fluids of smokers) [158]. Lockwood showed that CatB is redox buffered by GSH/GSSG ratio. GSSG could reverse GSH-dependent activation of CatB, while dihydrolipoic acid (DHLA) was identified as a potent activator [159]. When assessed in cysteine:cystine redox buffers (pH 6.0–7.0), CatB was active over a broad redox potential range, and authors suggested that CatB may be redox regulated in vivo at neutral pH [160]. Comparably, cathepsins B, K, L and S were capable of processing thyroglobulin under conditions mimicking the extracellular oxidizing environment of the thyroid follicle lumen (redox potential of −150 mV and neutral pH 7.2) [100]. Also, despite a significantly higher oxidative stress index (OSI) in current smokers compared to non-smokers, an increase of CatS activity was found in lung tissue of smokers [76]. Addition of N-acetyl cysteine or GSH led to a partial recovery of CatS activity following incubation with CSE, H_2_O, and ONOO^−^ [77]. A decrease in the inactivation rate by oxidized glutathione (GSSG) was observed for human CatK in the presence of a peptide substrate, suggesting that the latter has a partial protective effect against inactivation. However, k_obs_ (observed first-order rate constant for inactivation) values did not depend on the peptidyl substrate concentration in the presence of chondroitin-4-sulfate (C-4S) and led to improved stability of CatK against inactivation (Figure 5). Taking into account that human CatK has a binding exosite to C-4S that is essential for collagen hydrolysis, but not for peptide hydrolysis [161,162], the data suggest that the binding of C-4S to its exosite may favor the overall cathepsin K stability towards inactivation by oxidants.

#### 3.2.4. Carbonylation of Cysteine Cathepsins

During carbonyl stress, an accumulation of reactive carbon species occurs. These carbon species, such as reactive aldehydes, may oxidize carbohydrates or lipids, forming carbonyl-containing intermediate products which in turn can lead to the carbonylation of adjacent proteins. Both reactive aldehydes and carbonyl-containing proteins inhibit papain and cathepsins B and L by glycation of Cys25 of the active site [163]. Nevertheless, spectral analysis by circular dichroism supported that oxidation by CSE does not alter the overall structure of CatS, despite an increase of carbonylation [77]. Acrolein (Figure 4b), which is known to react with thiolate by forming a highly-stable Michael addition adduct, and in a lesser extent formaldehyde (Figure 4c), inactivated human cathepsins B, L, S and K in a concentration-dependent manner. Conversely to that observed in the presence of hydrogen peroxide, CatL is apparently less vulnerable than cathepsins B, S and K to inactivation by these two major chemicals of cigarette smoke. Again, data support that the difference in chemical reactivity depends on subtle differences in the environment of Cys25 within the active site of these closely related enzymes. The reaction of papain with glyoxal leads to the formation of S-carboxymethylcysteine, whereas formation of inter-molecular cross-links was observed in the presence of methylglyoxal [163]. During oxidative stress, one of the preferential targets of ROS are low-density lipoprotein (LDL). Previous studies have shown that oxidized-LDL (oxLDL), its lipid peroxidation by-product 4-hydroxy-2nonenal (HNE) or 4-HNE-modified LDL, may also inactivate CatB [164,165,166,167]. Accordingly, endosomal/lysosomal accumulation of oxLDL conducted to the intracellular inactivation of both cathepsins B and Cat L in cellulo [166,168,169,170]. Also, it was reported that other by-products of lipid peroxidation (e.g., phosphatidylcholine hydroxy alkenal and malondialdehyde) impair the activity of CatB and CatL [171,172]. During lung inflammatory episodes, ROS and RNS react with unsaturated fatty acids to form nitrated fatty acids (NFAs); subsequently, NFAs could downregulate the expression of CatS and specifically react with Cys25 via an electrophilic S-alkylation, thereby impairing its elastinolytic activity and its ability to promote emphysema under certain circumstances [173]. 

## 4. Conclusions

In the present review, we have uncovered the impact of oxidation on the catalytic activity of cysteine cathepsins and summarized the current knowledge about still somewhat-understood oxidation mechanisms of nucleophilic Cys25 of the active site. Nevertheless, such awareness mainly corresponds to in vitro results. In vivo evidence about the cellular consequences of cathepsin oxidation during oxidative stress induced by cigarette smoke or exposure to a polluted atmosphere (e.g., ozone, diesel particles) are lacking. Accordingly, consequences of Cys25 oxidation on signaling pathways in which cathepsins participate remain almost a terra incognita field of survey. Under pathophysiological conditions, it has been proposed that the oxidative modification of proteins may represent a distinctive mechanism involved in cell signaling and repair responses to tissue injury [174]. There is also rising evidence that reversible cysteine-targeted modifications of proteins (e.g., S-thiolation and S-nitrosylation) correspond to additional regulatory mechanisms that could even outnumber those that are regulated by phosphorylation [175]. Besides the direct use of hydrogen peroxide by cells as a second messenger for signal transduction and signal amplification, sulfenic acid formation could be also a specific and key post-translational regulatory mechanism of protein function [176,177]. Under these circumstances, analysis of the consequences of cathepsin oxidation on cell signaling pathways, which can be switched on/off in a redox-dependent manner, is a promising avenue of investigation. Such exploration could contribute to a better understanding of pathophysiological mechanisms involved in the onset of lung fibrosis, COPD, or other diseases closely related to oxidative stress.

## Figures and Tables

**Table 1:** Specific probes used for detection and labeling of sulfenic acid and sulfinic acid.

Target	Warhead	Tag/Detection Method	Probes (Usual Names)	Refs
Sulfenic acid	1,3-cyclohexanedione (dimedone derivatives)	7-methoxycoumarin	DCP-MCC	[113]
		Isatoic acid	DCP-MAB	[113]
		Fluorescein	DCP-FL1, DCP-FL2	[114]
		Rhodamine	DCP-Rho, DCP-Rho2	[114,115]
		Biotin	DCP-Bio1, DCP-Bio2, DCP-Bio3, Biotin dimedone	[114,115,116]
		alkyne–azide “click” chemistry	DAz-1, DAz-2	[117,118,119]
		alkyne–azide “click” chemistry	DYn-1, DYn-2	[120]
	1,3-cyclopentanedione	Biotin	BP1	[121]
	Linear β-ketoester	alkyne–azide “click” chemistry	Alk-β-KE	[122]
	Bicyclo[6.1.0]nonyne	Biotin	BCN-Bio	[123]
	7-chloro-4-nitrobenzo-2-oxa-1,3-diazole	Absorbance (347 nm)	NBD-Cl	[124]
	Norbornene	Biotin	Norb-Bio	[125]
		alkyne–azide “click” chemistry	Norb-yne	[125]
Sulfinic acid	2-nitroso terephthalic acid	Biotin	NO-Bio	[126]

## Abbreviations

C-4S	Chondroitin-4-sulfate
Cat	Cathepsin
CF	Cystic fibrosis
COPD	Chronic obstructive pulmonary disease
CSE	Cigarette smoke extract
ECM	Extracellular matrix
GAG	Glycosaminoglycan
GSH	Reduced glutathione
GSNO	S-nitrosoglutathione
GSSG	Oxidized glutathione
H_2_O_2_	Hydrogen peroxide
HOCl	Hypochlorous acid
HNO	Nitroxyl
IFN	Interferon
IL	Interleukin
LDL	Low-density protein
MHC	Major histocompatibility complex
MMP	Matrix metalloproteinase
NO	Nitric oxide
ONOO^−^	Peroxynitrite
RNS	Reactive nitrogen species
ROS	Reactive oxygen species
SP	Surfactant protein
TGF	Transforming growth factor
Enzymes	Cathepsin B, (EC 3.4.22.1), cathepsin C (EC 3.4.14.1), cathepsin H (EC 3.4.22.16), cathepsin K (EC 3.4.22.38), cathepsin L (EC 3.4.22.15), cathepsin S (EC 3.4.22.27), papain (EC 3.4.22.2)
